# Comment on the article ‘Protein crystal lattices are dynamic assemblies: the role of conformational entropy in the protein condensed phase’

**DOI:** 10.1107/S2052252518006267

**Published:** 2018-05-11

**Authors:** Massimo Nespolo

**Affiliations:** a CNRS, CRM2, Université de Lorraine, Nancy, F-54000, France

**Keywords:** protein crystals, crystal lattices

## Abstract

A comment is given on Dimova & Devedjiev [*IUCrJ* (2018), **5**, 130–140].

In a recent article by Dimova & Devedjiev (2018 ▸), the word ‘lattice’ occurs 32 times, and the term ‘crystal lattice’ 23 times. Actually, none of these occurrences concern the *lattice* but the *structure*. The distinction is of paramount importance because a lattice is an abstract mathematical concept that expresses the periodicity of the atomic distribution in the crystal space; the latter is the crystal structure. The confusion between these two fundamental concepts may lead to serious misunderstandings, like the term ‘polar lattice’ (page 136). A lattice being always centrosymmetric (in an odd-dimensional space), it can never be polar, whereas the crystal structure can. Furthermore, the term ‘polar lattice’ is used to indicate a completely different concept: the ancestor of the reciprocal lattice (Nespolo & Souvignier, 2010 ▸).
